# Supplementary material for: Probing Majorana localization of a phase-controlled three-site Kitaev chain with an additional quantum dot
Source: Nat Commun. 2026 Feb 3;17:2313. doi: 10.1038/s41467-026-68897-0 (PMC12976129; doi:10.1038/s41467-026-68897-0)
Supplement: Supplementary file 1 — Supplementary Information [file 41467_2026_68897_MOESM1_ESM.pdf]

# SUPPLEMENTARY INFORMATION

## Probing Majorana localization of a phase-controlled three-site Kitaev chain with an additional quantum dot

Alberto Bordin,[1,*] Florian J. Bennebroek Evertsz',[1,*] Bart Roovers,[1,*] Juan D. Torres Luna,[1,*] Wietze D. Huisman,[1] Francesco Zatelli,[1] Grzegorz P. Mazur,[1] Sebastiaan L. D. ten Haaf,[1] Ghada Badawy,[2] Erik P. A. M. Bakkers,[2] Chun-Xiao Liu,[1] Ruben Seoane Souto,[3] Nick van Loo,[1] and Leo P. Kouwenhoven[1,†]

[1] *QuTech and Kavli Institute of NanoScience, Delft University of Technology, Delft, The Netherlands*
[2] *Department of Applied Physics, Eindhoven University of Technology, Eindhoven, The Netherlands*
[3] *Istituto de Ciencia de Materiales de Madrid (ICMM), Consejo Superior de Investigaciones Científicas (CSIC), Sor Juana Inés de la Cruz 3, 28049 Madrid, Spain*

[*] These authors contributed equally to this work.

[†] l.p.kouwenhoven@tudelft.nl

## THEORETICAL MODEL

### Spinless model

We extend the spinless Kitaev chain model to include an additional quantum dot as follows:

$$H = H_N + H_{\mathrm{AD}} + H_{\mathrm{tunnel}}, \tag{1}$$
$$H_{\mathrm{AD}} = (\mu_0 - \mu_{\mathrm{offset}}) c_0^\dagger c_0, \tag{2}$$
$$H_{\mathrm{tunnel}} = t_d c_0^\dagger c_1 + \Delta_d c_0^\dagger c_1^\dagger + U_{\mathrm{nl}} n_0 n_1 + \mathrm{h.c.}, \tag{3}$$

where $H_N$ is given by Eq. 1 in the main text. The additional quantum dot is described by a single level $c_0$ with chemical potential $\mu_0$, which is connected to $V_{\mathrm{AD}}$ via the lever arm $\alpha_0$: $(\mu_0 - \mu_{\mathrm{offset}}) = -e\alpha_0 \delta V_{\mathrm{AD}}$. In the experimental data, the $V_{\mathrm{AD}}$ range is not perfectly centred around the AD resonance, therefore, we allow for a small chemical potential shift $\mu_{\mathrm{offset}}$ in the model. The coupling between AD and D1 is mediated by a normal tunneling term $t_d$ and a phenomenological superconducting term $\Delta_d$. Finally, we consider a non-local Coulomb interaction $U_{\mathrm{nl}}$ between dots AD and D1. $n_i = c_i^\dagger c_i$ is the number operator at site $i$.

### Spinful model

Complementing the spinless model, where the only source of imperfection for the Majoranas is detuning from the sweet spot, we study the spinful model, that considers additional sources of imperfection [1, 2]. In particular, the spinful model includes three additional variables: the Zeeman energy $E_Z$, a local charging energy $U$ on every site, and the spin-orbit angle $\theta$. For simplicity, we fix the Zeeman and local charging energies to $E_{\mathrm{Z}} = 200\,\mu\mathrm{eV}$ and $U = 3\,\mathrm{meV}$. The Hamiltonian for the spinful Kitaev chain coupled to an additional quantum dot is:

$$H = H_{\mathrm{QDs}} + H_{\mathrm{AD}} + H_{\mathrm{tunnel}}, \tag{4}$$

where the elements of the Hamiltonian are given by:

$$\begin{aligned} H_{\mathrm{QDs}} = \sum_{i=1}^{N} & \left[(\mu_i + E_Z)\, n_{i\uparrow} + (\mu_i - E_Z) n_{i\downarrow}\right] + U n_{i\uparrow} n_{i\downarrow} + t_i^{\mathrm{sc}} \left[c_{i\uparrow}^\dagger c_{i+1\uparrow} + c_{i\downarrow}^\dagger c_{i+1\downarrow}\right] + t_i^{\mathrm{so}} \left[c_{i\uparrow}^\dagger c_{i+1\downarrow} - c_{i\downarrow}^\dagger c_{i+1\uparrow}\right] \\ & + \Delta_i^{\mathrm{sc}} \left[c_{i\uparrow}^\dagger c_{i+1\uparrow}^\dagger + c_{i\downarrow}^\dagger c_{i+1\downarrow}^\dagger\right] + \Delta_i^{\mathrm{so}} \left[c_{i\uparrow}^\dagger c_{i+1\downarrow}^\dagger - c_{i\downarrow}^\dagger c_{i+1\uparrow}^\dagger\right] + \mathrm{h.c.}, \end{aligned} \tag{5}$$

$$H_{\mathrm{AD}} = (\mu_0 + E_Z - \mu_{\mathrm{offset}})\, n_{0\uparrow} + (\mu_0 - E_Z - \mu_{\mathrm{offset}}) n_{0\downarrow} + U n_{0\uparrow} n_{0\downarrow}, \tag{6}$$

$$H_{\mathrm{tunnel}} = t_d^{\mathrm{sc}} \left[c_{0\uparrow}^\dagger c_{1\uparrow} + c_{0\downarrow}^\dagger c_{1\downarrow}\right] + t_d^{\mathrm{so}} \left[c_{0\uparrow}^\dagger c_{1\downarrow} - c_{0\downarrow}^\dagger c_{1\uparrow}\right] + \Delta_d^{\mathrm{sc}} \left[c_{0\uparrow}^\dagger c_{1\uparrow}^\dagger + c_{0\downarrow}^\dagger c_{1\downarrow}^\dagger\right] + \Delta_d^{\mathrm{so}} \left[c_{0\uparrow}^\dagger c_{1\downarrow}^\dagger - c_{0\downarrow}^\dagger c_{1\uparrow}^\dagger\right] \tag{7}$$

$$+ U_{\mathrm{nl}}(n_{0\uparrow} n_{1\uparrow} + n_{0\downarrow} n_{1\downarrow} + n_{0\uparrow} n_{1\downarrow} + n_{0\downarrow} n_{1\uparrow}) + \mathrm{h.c.} \tag{8}$$

Inside the chain, the spin-conserving and spin-orbit interactions

$$t_i^{\mathrm{sc}} = \tau_i \cos(\theta/2), \quad t_i^{\mathrm{so}} = \tau_i \sin(\theta/2), \tag{9}$$
$$\Delta_i^{\mathrm{sc}} = \eta_i \sin(\theta/2), \quad \Delta_i^{\mathrm{so}} = \eta_i \cos(\theta/2), \tag{10}$$

where $\tau_i$ is the amplitude of the hopping, $\eta_i$ is the amplitude of the pairing, and $\theta$ is the same spin-orbit angle as inside the Kitaev chain.

In contrast to the spinless model where the sweet spot is $\mu_i = 0$ and $t = \Delta$, the spinful model requires tuning of the system parameters. For a fixed $E_Z$ and $U$, we numerically find the sweet spot $\mu_i^*(\theta)$ and $\eta_i^*(\theta)$ for $0 < \theta < \pi$ for $i > 0$. To match the tuning procedure of a three site Kitaev chain indicated in Fig. 2, we first find the sweet spots for the left PMM with $\tau_1 = 12\mu\mathrm{eV}$ and the right PMM with $\tau_2 = 9\mu\mathrm{eV}$ separately. We then fix the left PMM to the sweet spot, and bring the right dot to the corresponding value.

We remark that this model is highly simplified to restrict the number of fitting parameters. For instance, it does not explicitly include the states in the hybrid section, and it assumes uniform $U$ and $\theta$ along the chain. More complete models are worth exploring in future studies.

## FIT OF THE QUANTUM DOT TEST

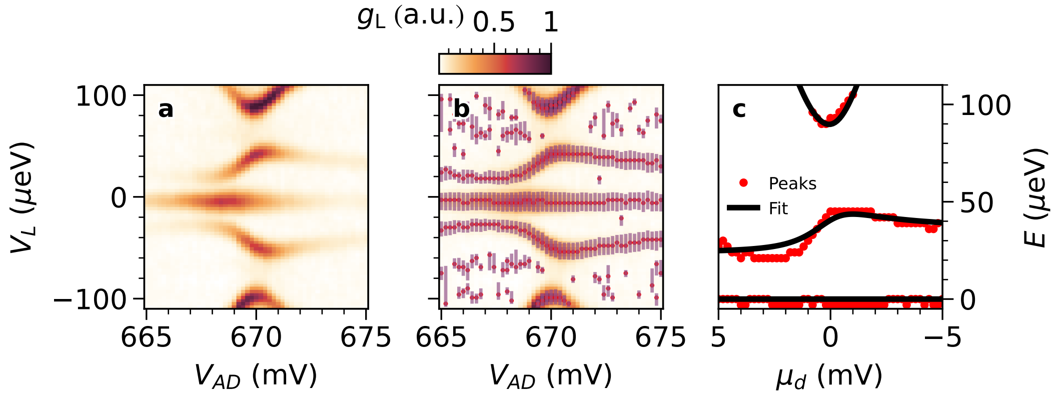


Fig. S1. **Illustration of the fitting procedure of the experimental data.** **a.** Normalized conductance spectrum from Fig. 4c. **b.** Extraction of the peak positions and width from the experimental data in panel **a**. **c.** Peaks yelding continuous lines (red dots) and the corresponding fit using Eq.(1) (black lines).

In order to systematically fit the experimental data presented in the main text, we use the following procedure:

1. For every value of $V_{\mathrm{AD}}$, we extract the peaks in the differential conductance using the `find_peaks` method from `scipy` as shown in Fig. S1 (b).
2. We disregard the peaks that appear from the noise by identifying the most continuous lines in the signal as shown in Fig. S1 (b,c) using the code reported in the linked repository [3].
3. We label the extracted peaks as $\mathcal{E}_{ij}$ where the index $i$ describes the level and the index $j$ describes the voltage of the additional dot.
4. For a given set of microscopic parameters we compute the eigenvectors and eigenvalues of each model and obtain the excitation energy spectrum $E_{ij}(\mathbf{x})$ where the indexes $i$ and $j$ are the same as before. We keep only transitions that are visible from the additional quantum dot side, that is, transitions such that $|\langle\psi_i|c_0|\psi_0\rangle| > \epsilon$ where $|\psi_0\rangle$ is the ground state, $|\psi_i\rangle$ are the excited states of the opposite parity and $\epsilon \sim 10^{-3}$ is a threshold.
5. Finally, we use the optimiser `differential_evolution` [4] to find the minimum of the cost function:

$$\mathcal{C} = \sum_{i,j} |\mathcal{E}_{ij} - E_{ij}(\mathbf{x})|^2 . \tag{11}$$

While it is possible to fit the data for all the parameters in the model, this approach is susceptible to over-fitting given the large amount of parameters. Therefore, we fix the chain parameters using the experimental configuration as a guide and only optimise for the unknown parameters. Although this limits the quality of the fit, it guarantees that we do not overfit the data since the role of each interaction is well-defined. We fix the chemical potentials $\mu_i$ and the interactions within the chain $|t_i| = |\Delta_i|$, as shown in the linked repository [3]. For the spinless model, we fit the following microscopic parameters:

$$\mathbf{x} = (U_{\mathrm{nl}}, t_d, \Delta_d, \alpha_0, \mu_{\mathrm{offset}}). \tag{12}$$

In the case of the spinful model, we also optimise for $\theta$. The bounds for all the optimisation parameters are sufficiently large so that we do not guide the system to a particular value.

### A. Spinless model

We fit the datasets presented in Fig. 4 using the spinless model. The parameters extracted from the fitting are listed in Table I and the corresponding transport simulations are shown in Fig. S2 and S3. We observe that the optimal solution yields systematic results for the strength of the different coupling parameters. Namely, we extract $t_d \sim 50\,\mu\mathrm{eV}$, $\Delta_d \sim 30\,\mu\mathrm{eV}$, and $U_{\mathrm{nl}} \sim 20\,\mu\mathrm{eV}$.

| | Fixed parameters | | | | | | | Optimisation parameters | | | | |
|---|---|---|---|---|---|---|---|---|---|---|---|---|
| Experiment | $\mu_1$ | $\mu_2$ | $\mu_3$ | $\Delta_1$ | $t_1$ | $\Delta_2$ | $t_2$ | $U_{\text{nl}}$ | $t_d$ | $\Delta_d$ | $\alpha_0$ | $\mu_{\text{offset}}$ |
| Fig.S2(a) | 0 | 0 | | 12 | 12 | | | 26 | 50 | 30 | 0.05 | 18 |
| Fig.S2(b) | 26 | 0 | | 12 | 12 | | | 17 | 52 | 29 | 0.06 | 6 |
| Fig.S2(c) | 0 | 26 | | 12 | 12 | | | 26 | 46 | 30 | 0.05 | 20 |
| Fig.S2(d) | 23 | 26 | | 12 | 12 | | | 20 | 58 | 32 | 0.06 | -6 |
| Fig.S3(a) | 0 | 0 | 0 | 12 | 12 | 9 | 9 | 28 | 50 | 28 | 0.05 | 17 |
| Fig.S3(b) | 26 | 0 | 0 | 12 | 12 | 9 | 9 | 21 | 51 | 28 | 0.07 | -11 |
| Fig.S3(c) | 0 | 26 | 0 | 12 | 12 | 9 | 9 | 21 | 51 | 30 | 0.04 | 8 |
| Fig.S3(d) | 0 | 0 | 26 | 12 | 12 | 9 | 9 | 27 | 51 | 28 | 0.05 | 15 |
| Fig.S3(e) | 23 | 26 | 22 | 12 | 12 | 9 | 9 | 24 | 57 | 30 | 0.06 | -18 |

TABLE I. **Optimised parameters for the spinless three-site chain.** All parameters are given in µeV except for $\alpha$, which is dimensionless. The chemical potentials and interactions inside the chain are fixed according to the experimentally extracted values. We optimise five parameters listed in the rightmost columns.

## B. Spinful model

In Table II we report the results of the fitting spinful model corresponding to the sweet-spot shown in Fig. 4 (d, h).

| | Fixed parameters | | | | Optimisation parameters | | | | | | Sweet spot parameters | | | | |
|---|---|---|---|---|---|---|---|---|---|---|---|---|---|---|---|
| Experiment | $U$ | $E_Z$ | $\tau_1$ | $\tau_2$ | $\theta/\pi$ | $U_{\text{nl}}$ | $\tau_0$ | $\eta_0$ | $\alpha_0$ | $\mu_{\text{offset}}$ | $\mu_1(\theta)$ | $\mu_2(\theta)$ | $\mu_3(\theta)$ | $\eta_1(\theta)$ | $\eta_1(\theta)$ |
| 4 2site | 3000 | 200 | 12 | | 0.18 | 14 | 69 | 87 | 0.05 | 20 | 197 | 197 | | 35 | |
| 4 3site | 3000 | 200 | 12 | 9 | 0.21 | 13 | 66 | 72 | 0.05 | 19 | 198 | 198 | 199 | 31 | 24 |

TABLE II. **Optimised parameters for the spinful three-site chain.** The units are $\mu$eV except for $\alpha$ and $\theta$. We fix the Zeeman energy, Coulomb interaction, and hopping amplitudes inside of each chain. We optimize for the interaction between the external dot and the Kitaev chain. We also optimize the spin-orbit angle $\theta$ which requires the chemical potentials and the strength of the superconducting pairing inside of the chain to be at the sweet spot.

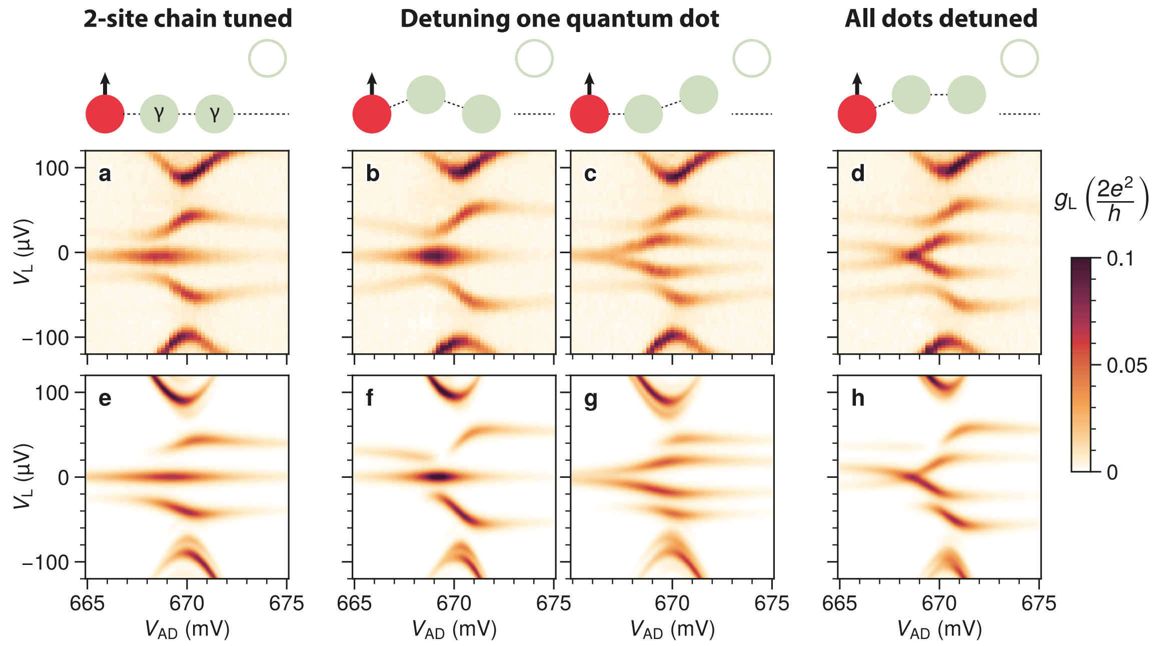


Fig. S2. **QD-test for sweet spot A, showcasing all detuning configurations for the left two-site chain.** Here, we report the measured conductance spectra from the left lead for the QD-test performed on the left two-site chain, formed by D1 and D2, with D3 5 mV off-resonance. **a.** Replot of Fig. 4c, with the two-site Kitaev chain tuned to the sweet spot. **b,c.** QD-test with either the left QD (D1) or the right QD (D2) of the two-site chain detuned by 2 mV. Detuning D1 (D2) shifts the wavefunction of $\gamma_1$ ($\gamma_{2N}$) towards D2 (D1). Notably, only the latter results in an observable splitting of the ZBP, as the QD-test is mostly sensitive to a Majorana overlap on D1. **d.** Replot of Fig. 4a, with both D1 and D2 detuned by 2 mV. **e-h.** Corresponding theoretical simulations of the differential conductance for the QD-test. The calculations are performed following the rate-equation approach [1], assuming a finite temperature of $k_B T = 3\,\mu$eV and a finite coupling to the normal leads of $\Gamma_L = \Gamma_R = 3.5\,\mu$eV. The parameters of the simulation are listed in Table I.

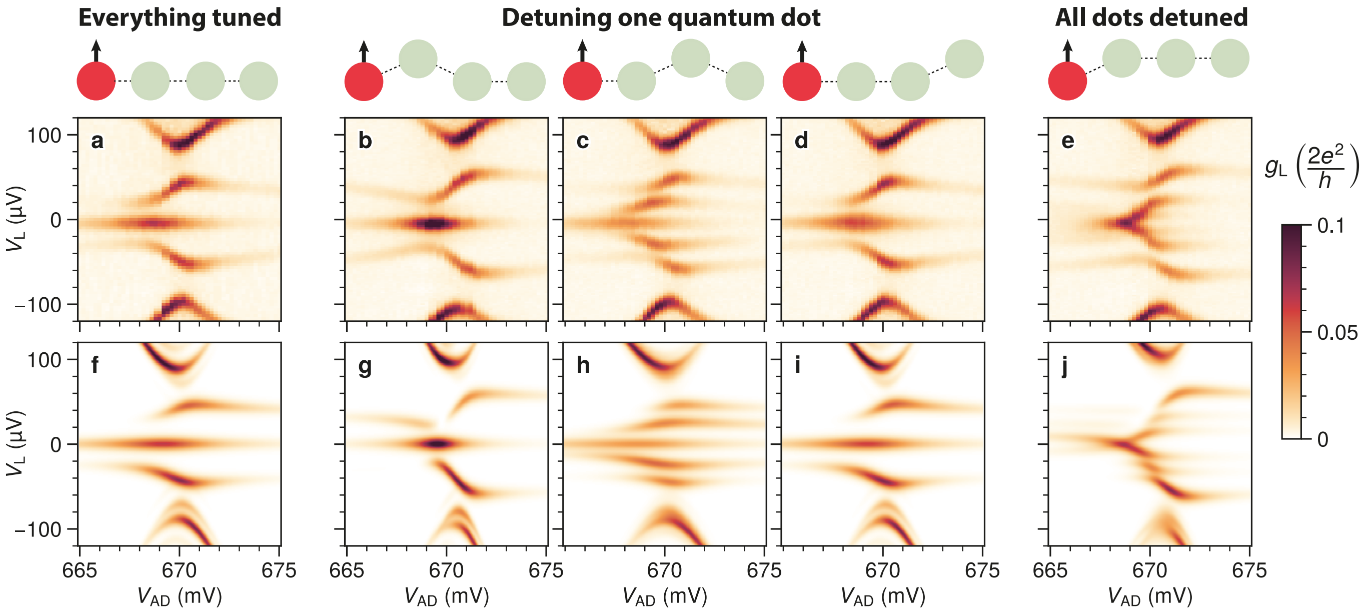


Fig. S3. **QD-test for sweet spot A, showcasing all detuning configurations for the three-site Kitaev chain.** Here, we report the measured conductance spectra from the left lead for the QD-test performed on the three-site Kitaev chain. **a.** Replot of Fig. 4g, with the three-site chain tuned to the sweet spot. **b-d.** QD-test with respectively D1, D2, or D3 detuned by 2 mv. A detuning of one of the outer QDs (D1 or D3) shifts the wavefunction of $\gamma_1$ or $\gamma_{2N}$ towards the middle QD (D2) [5]. A detuning of D2, in contrast, enhances the localization of $\gamma_1$ and $\gamma_{2N}$ on D1 and D3 respectively [2]. In all cases, no detectable Majorana wavefunction overlap is induced on D1. **e.** Replot of Fig. 4e, with all three QDs detuned by 2 mV. **f-j.** Corresponding theoretical simulations of the differential conductance for the QD-test. The calculations are performed following the rate-equation approach [1], assuming a finite temperature of $k_B T = 3\,\mu$eV and a finite coupling to the normal leads of $\Gamma_L = \Gamma_R = 3.5\,\mu$eV. The parameters of the simulation are listed in Table I.

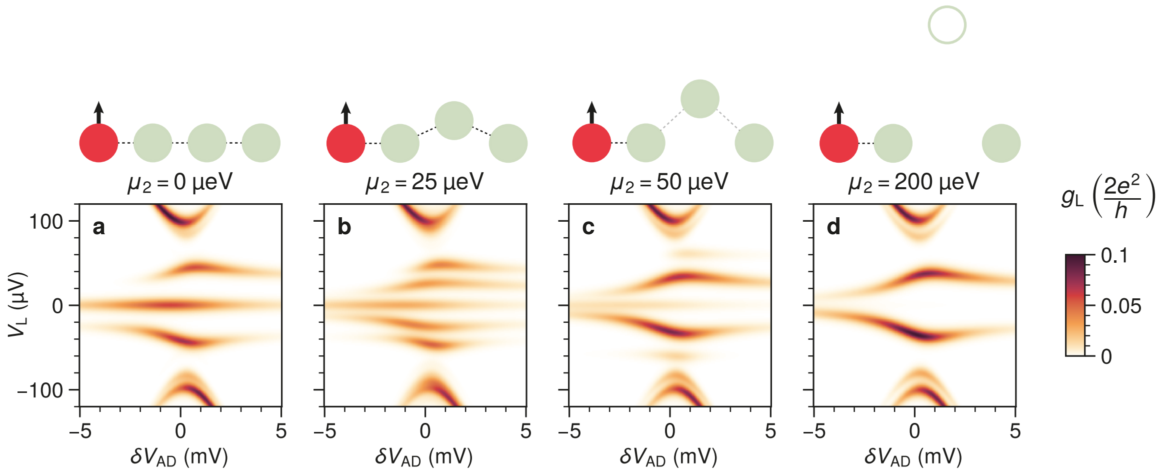


Fig. S4. **Theoretical simulations of the QD-test for varying D2 detunings.** Here, we present theoretical simulations of the QD-test performed on a three-site Kitaev chain for varying detunings of D2. From left to right, the panels illustrate the evolution from a perfectly tuned three-site chain, to a regime where the strongly detuned middle dot (D2) effectively splits the three-site chain in half. The simulations are performed using the same coupling amplitudes as listed in Table I. **a.** QD-test with the three-site Kitaev chain tuned to the sweet spot. **b-d.** QD-test with D2 detuned by 25 µeV, 50 µeV, and 200 µeV respectively, corresponding to roughly one, two, and eight times the excitation gap of the individual two-site chains. The evolution from a three-site chain to an effectively split chain, is reflected in the number and visibility of (excited) states within the voltage bias window of 0 µeV to 100 µeV.

## FIT OF THE PHASE DEPENDENCE OF THE THREE SITE KITAEV CHAIN

In Fig. 2 (e-g) in the main text we show the distribution of the phase shift $\varphi_0$ at zero out-of-plane field $B_z = 0$. To fit the experimental data, we consider the Kitaev chain Hamiltonian from Eq. 1 of the main text for a three site chain exactly at the sweet spot, that is, we set $\mu_i = 0$, $t_1 = |\Delta_1|$ and $t_2 = |\Delta_2|e^{i(\varphi-\varphi_0)}$. Under these conditions, we diagonalise Eq. 1 analytically and obtain six eigenvalues. Because of particle-hole symmetry and because the chain is perfectly tuned, there are only two non-trivial eigenvalues:

$$E_{\pm,\varphi}(\Delta_1, \Delta_2, \varphi_0) = \sqrt{2(\Delta_1^2 + \Delta_2^2) \pm 2\sqrt{\Delta_1^4 - 2\Delta_1^2\Delta_2^2\cos(\varphi - \varphi_0) + \Delta_2^4}}. \tag{13}$$

We calculate the phase difference as $\varphi = B/T_\varphi$ where $T_\varphi$ is the period of oscillation. To systematically extract $\varphi_0$, we use a fitting procedure similar to that in Eq. (11), but in this case, we compare the analytical expressions of Eq. (13) with the lowest two excited states from the conductance measurements. We minimize the following cost function

$$\mathcal{C}' = \sum_{i=\pm}\sum_{B} \left|\mathcal{E}_{i,B} - E_{i,B}(\Delta_1, \Delta_2, \varphi_0, T_\varphi)\right|^2. \tag{14}$$

Here, $B$ runs over the measured range of out-of-plane fields and $i = \pm$ runs over the two excited states of the Kitaev chain. The optimization parameters are the induced gaps $\Delta_1$ and $\Delta_2$, phase shift $\varphi_0$, and the period $T_\varphi$.

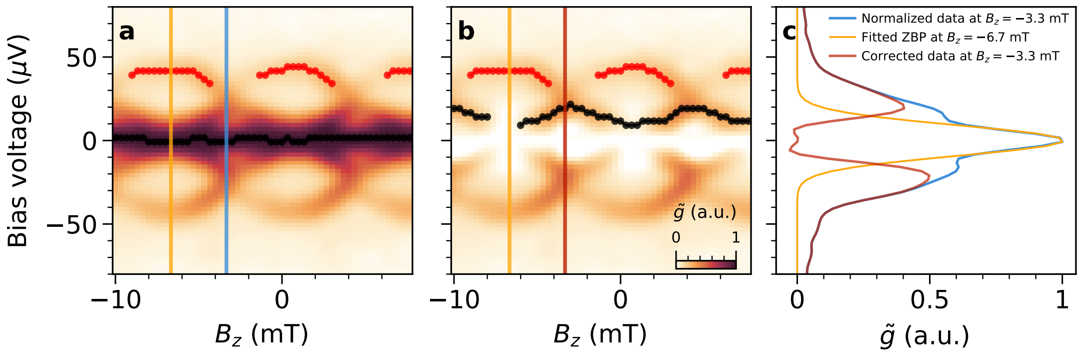


Fig. S5. **Post processing of the conductance data used for $\varphi_0$ extraction. a** Conductance measurement after applying a Gaussian filter with $\sigma = 1$ and normalizing each bias scan. Yellow line corresponds to the position of the sharpest ZBP in the range of $B$. Blue line corresponds to the cut shown in panel (c). **b** Data after removing the sharpest ZBP found in the scan. This procedure involves fitting all ZBPs using a Gaussian function and choosing the one with the smallest standard deviation. Red line corresponds to the corrected data shown in panel (c). **c** Comparison of the data before (blue line) and after the ZBP subtraction (red line). Note that the reference fitted ZBP (yellow line) is taken at a different $B$.

In panel Fig. S5 (a) we observe that the extracted peaks of the first excited state (black dots) are indistinguishable from the zero-bias peak. Since we are not interested in the zero-bias peak, we post-process the data to easily identify the features of the first excited state. The post processing steps are:

1. To maximize the signal, we add the local conductance from the left and right leads.
2. We normalize each bias scan, and apply a Gaussian filter with $\sigma = 1$ to the conductance.
3. We identify the bias scan with the sharpest zero-bias peak by fitting the signal around zero bias with a Gaussian.
4. We subtract the fitted Gaussian from all bias scan.
5. Finally, we extract the peaks from the processed scans.

We illustrate the post-processing protocol in Fig. S5. The original signal is shown in panel (a) and the post-processed signal is shown in panel (b). We observe that the peaks of the first excited state (black dots) are easily identified by the peak extraction routine. In panel (c) we compare the original data (blue solid line) with the corrected data after the ZBP subtraction (red line). We choose a cut shown by the red line to illustrate that subtracting the zero-bias peak enhances the features of the first excited state.

The results of the fit are presented in Fig. S6. In panels (a-k), we show the resulting fit of each sweet spot configuration. Overall, we observe that the periodicity of the spectrum is captured by the model, and for some cases,

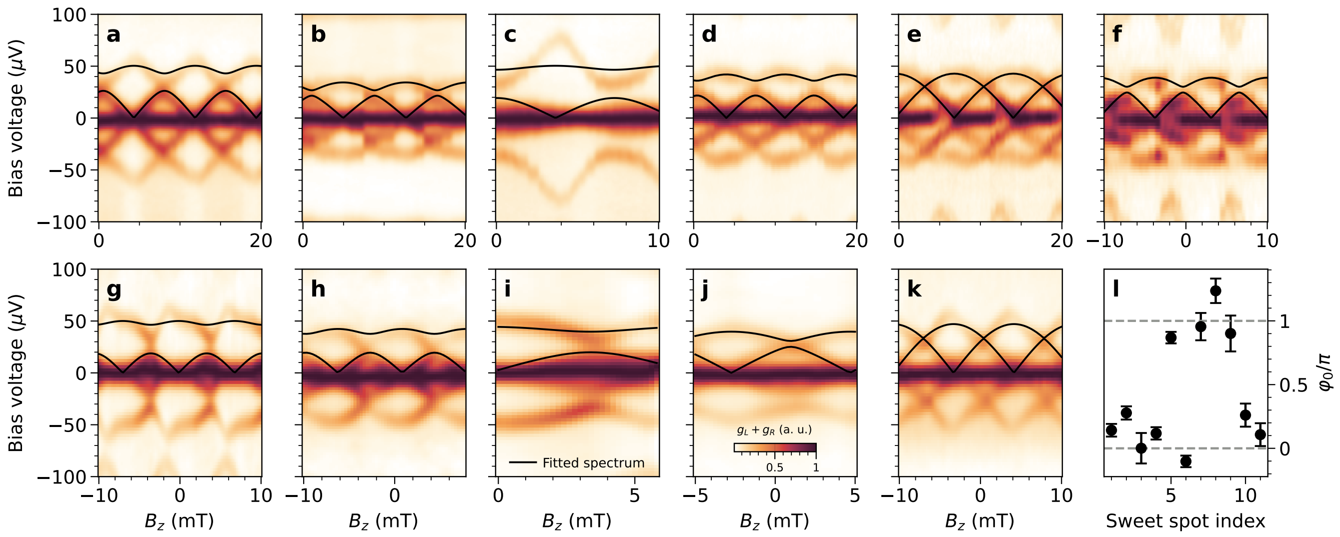


Fig. S6. **Fitting of the phase shift $\varphi_0$ at $B_z = 0$ for different sweet spot configurations. a-k.** Normalized conductance measurements as a function of out-of-plane field $B_z$ in units of the period $T_\varphi = 7.5$ mT with the fit result superimposed as solid black lines. We use the sum of left and right conductances for all plots. **l.** Distribution of extracted $\varphi_0$ for all the measured sweet spots. This is in agreement with distribution shown in Fig. 2 (g). The error bars are a sum of the fit error from the covariance matrix and the experimental uncertainty in the magnetic field. The large discrepancy in error bar size between the first six and final five measurements is due to a change in magnet power supply.

such as panels (a) and (b), the fitted spectrum agrees with the measured data. In other cases, such as panels (c), (g), and (h), we observe that the second excited state becomes asymmetric because the ABS is close in energy. Since our model does not include the ABS, the fit of the second excited state is less accurate in these cases. However, the periodicity of both excited states is well captured by Eq. 1 of the main text. In panel (l), we show the distribution of phase shifts $\varphi_0$ for all the sweet spots. We observe that the resulting distribution shows two clusters of points around 0 and $\pi$.

| Dataset | $\Delta_1$ ($\mu$eV) | $E_{\Delta_1}$ ($\mu$eV) | $\Delta_2$ ($\mu$eV) | $E_{\Delta_2}$ ($\mu$eV) | $\varphi_0/\pi$ | $E_{\varphi_0}$ | $E_B/T_\varphi$ | $T_\varphi$ (mT) | $E_{T_\varphi}$ (mT) |
|---|---|---|---|---|---|---|---|---|---|
| a | 21.513 | 0.343 | 13.121 | 0.344 | 0.142 | 0.030 | 0.020 | 7.551 | 0.071 |
| b | 10.778 | 0.360 | 13.369 | 0.509 | 0.278 | 0.035 | 0.020 | 7.751 | 0.087 |
| c | 23.274 | 0.584 | 9.656 | 0.915 | 0.001 | 0.112 | 0.022 | 7.276 | 0.501 |
| d | 17.994 | 0.231 | 10.881 | 0.240 | 0.118 | 0.024 | 0.021 | 7.172 | 0.052 |
| e | 15.107 | 1.994 | 15.158 | 2.023 | 0.869 | 0.018 | 0.021 | 7.253 | 0.042 |
| f | 12.301 | 0.233 | 15.057 | 0.276 | -0.102 | 0.014 | 0.022 | 6.905 | 0.063 |
| g | 9.412 | 0.442 | 23.179 | 0.295 | 0.955 | 0.048 | 0.049 | 6.896 | 0.202 |
| h | 9.739 | 0.316 | 18.804 | 0.310 | 1.236 | 0.022 | 0.047 | 7.075 | 0.110 |
| i | 9.941 | 0.565 | 19.861 | 0.417 | 0.901 | 0.109 | 0.049 | 7.487 | 0.800 |
| j | 15.384 | 0.479 | 12.574 | 0.484 | 0.261 | 0.018 | 0.046 | 7.426 | 0.183 |
| k | 16.468 | 1.357 | 16.801 | 1.406 | 0.108 | 0.013 | 0.045 | 7.447 | 0.063 |

TABLE III. **Fitted parameters of phase dependence.** The table shows the optimized parameters for each sweet spot: $\Delta_1$ and $\Delta_2$ in $\mu$eV, the phase $\varphi_0$ normalized by $\pi$, and the period $T_\varphi$ in mT. Errors $E_x$ for a parameter $x$ are shown in separate columns for each parameter. The error for the magnetic field $E_B$ is 0.15 mT for the first six measurements and 0.33 mT for the last five measurements. The error $E_B/T_\varphi$ includes the propagated error from the period fit. For all other parameters, the errors are calculated using the covariance matrix.

## DISCUSSION OF ALTERNATIVE EXPLANATIONS

Potential Majorana signatures in long hybrid nanowires [6–8] have been contested by the emergence of alternative explanations, such as fine-tuned ABSs [9], Kondo effects [10], or quasi-Majoranas [11]. For hybrid nanowires targeting the Lutchyn-Oreg adaptation of the Kitaev model [12, 13], it is difficult to rule out all alternative explanations since fine-tuned configurations can mimic the ZBP signatures originally attributed to Majoranas [9, 14].

Conversely, ruling out possible alternatives is considerably easier in QD-based Kitaev chains. This is due to a combination of reasons:

1. Artificial Kitaev chains rely on two simple elements – QDs and ABSs – combined together in an alternating array [1]. Every element is characterized by its local spectrum. Long Lutchyn-Oreg wires are instead mesoscopic systems, hence harder to control and characterize.

2. The spin flavour can be verified independently, element by element, with a dedicated field sweep.

3. The finite chain size translates into an overall discrete spectrum. Not just ZBPs, but every excited state can be identified and mapped to the theory model.

4. Artificial Kitaev chains are defined with dense gate arrays. This contingency provides many more tuning knobs, compared to Lutchyn-Oreg nanowires, to falsify possible interpretations.

Thanks to the four reasons above, all the popular alternative explanations to Lutchyn-Oreg Majorana signatures are inconsistent with the measured spectra in artificial Kitaev chain:

- **Fine-tuned ABSs.**
  Two- and three-site chain MBSs are, in-fact, a non-local ABS fine-tuned to a specific sweet-spot. They are tuned with charge stability diagrams and characterized completely by their spectrum (reasons 1 and 3). The spectral dispersion against any QD detuning makes them unique (reason 4), ruling out any other known combination of local ABSs.

- **Kondo effects.**
  Kitaev chains are formed at finite field (reason 2) ruling out any zero-field Kondo effect. In principle, it is possible to hit accidental degeneracies at finite field as well, which could produce a Kondo-like spin screening. Here, accidental degeneracies are ruled out by both the QD spectroscopy (reason 1) and field sweeps (reason 2).

- **Quasi-Majoranas.**
  Quasi-Majoranas are special non-topological MBSs localized at one end of a hybrid nanowire. Their wave-functions might overlap without hybridizing and might couple differently to a tunnel probe. This makes quasi-Majoranas difficult to exclude in Lutchyn-Oreg nanowires [11]. In artificial Kitaev chains, quasi-Majoranas are ruled out by the simultaneous appearance of ZBPs and expected excited states at both chain ends (reason 3).

- **Coulomb interactions.**
  It was recently shown that robust ZBPs can also appear at zero field in two-site Kitaev chains [15, 16]. Here, these are ruled out by the finite field (reason 2) and the excited states (reasons 3 and 4), which would exhibit one extra line in the spectrum [15]. Robust ZBPs at zero field are consistent with Majorana Kramers' pairs, as opposed to genuine poor man's Majoranas, and are enabled by a large on-site charging energy [16]. However, this scheme doesn't extend to three-site chains: zero-field ZBPs due to Majorana's Kramers pairs split when a third QD is added to the chain [16].
  In general, the role of Coulomb interactions would benefit from further investigation. As of now, it is understood in the zero-field limit (Majorana Kramers' pairs [16]) and the infinite-field limit (Kitaev chain) where they do not play a role since large Zeeman energies forbid double QD occupancy. In the intermediate regime, the model becomes more complicated [17]. Numerical simulations suggest that Coulomb interactions do not change the qualitative behaviour [17].

We stress that all the pieces of evidence listed above are not specific to this work, but have been consistently reproduced over the last two years in multiple artificial Kitaev chains defined in either InSb nanowires or InSbAs 2DEGs [5, 15, 18–23]. Here, we verify them once more and with an unprecedented level of accuracy (Figs. 2, 3, ED3, ED4, ED5, ED6, ED7, ED8) and reproducibility (Figs. 2, ED4, ED9, ED10, ED11, ED16).

On top of this, here we also introduce the QD-test (Figs. 4, ED1, ED2, ED12, ED13, ED14), which further rules out fine-tuned ABSs and quasi-Majoranas. In fact, both these options may result in unsplit ZBPs if probed by a standard tunnel probe. Instead, the QD-test is sensitive to local Majorana overlap even where standard tunnel spectroscopy

is not. This is particularly appreciated in Fig. 4b, where D2 detuning de facto creates a local ABS in D1: here, the QD-test resolves a clear ZBP splitting, where standard tunnel spectroscopy cannot [15, 18, 20]. We stress once more that the QD test is not necessary to rule out these alternatives, since QD characterization (reason 1) and the excited state dispersion to local and non-local detunings (reasons 3 and 4) also suffice, but it is a novel, elegant, and powerful solution.

In summary, the verifiability of artificial Kitaev chains, via reasons 1–4 and the newly added QD-test, provides converging evidence against known alternative explanations. Their repeated observation across devices and platforms further supports this interpretation and strengthens confidence in the measurements [5, 15, 18–23]. Together, these features indicate that artificial Kitaev chains offer a consistent route for Majorana physics in condensed-matter systems [24]. Shifting the paradigm, from Lutchyn-Oreg to QD-based Kitaev chains, also changes the relevant questions: now, the key query is no longer on the Majorananess, but rather on the usefulness. Since MBSs in few-site Kitaev chains are fine-tuned, hence non-topological, are they protected enough for practical applications? Existing estimates for modest lengths (five–six sites) are encouraging in terms of expected coherence times [22, 25] relative to other solid-state qubit platforms [26, 27], but direct time-domain validation via Rabi oscillations remains an important goal.

# Extended Data

- Figs. S7 to S9 concern the "sweet spot A" presented in the main text.
- Figs. S11 to S15 report the reproduction of the main results on another sweet spot ("sweet spot B").
- Figs. S16 and S17 compare sweet spots A and B.
- Fig. S18 reports all the measured phase dependences of 11 different sweet spots we found in the reported device. Different sweet spots differ in either hybrid or QD gate voltages, or both, and were measured to very different extents. QD-tests were performed only for the fourth and the eleventh sweet spot, corresponding to sweet spots A and B, respectively.

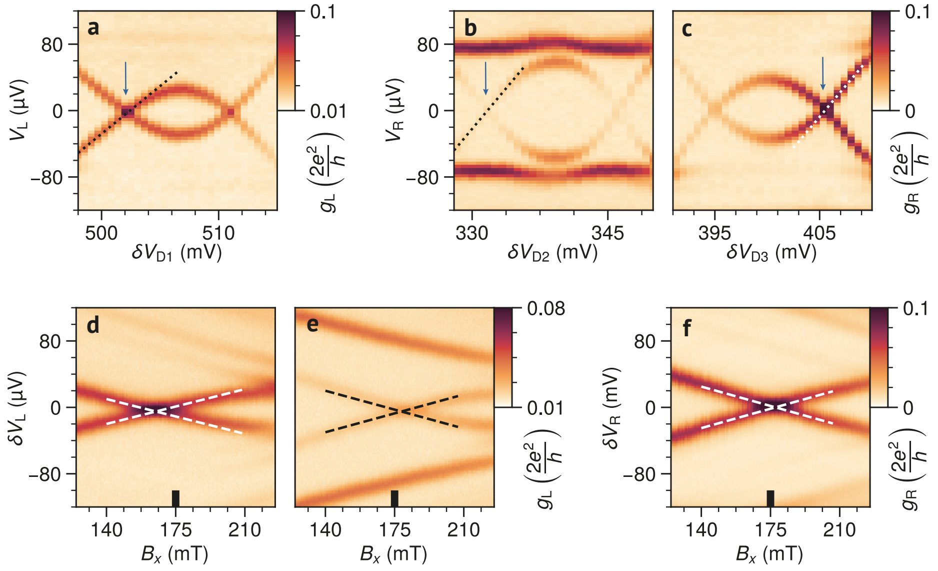


Fig. S7. **QD characterization of sweet spot A. a-c.** Conductance spectroscopy as a function of $V_{\mathrm{D1}}, V_{\mathrm{D2}}$ and $V_{\mathrm{D3}}$. Each panel shows a single QD orbital, measured with the other two quantum dots $\approx 5\,\mathrm{mV}$ off-resonance. The arrows highlight the used QD resonances for sweet spot A, corresponding to a spin configuration of: $\downarrow, \downarrow, \uparrow$ respectively. From the slope of the overlaid dotted lines, we estimate the lever arms $\alpha_1, \alpha_2, \alpha_3 = 0.011, 0.013, 0.015$, respectively, where $\delta\mu_n \equiv -e\alpha_n \delta V_{\mathrm{D}n}$. $B_x = 175\,\mathrm{mT}$. We note the presence of high-energy states at about $80\,\mathrm{\mu V}$, which we attribute to the other QDs set off-resonance. **d-f** Conductance spectroscopy as a function of $B_{\mathrm{x}}$, the field along the nanowire. Again, each panel is measured such that, at $175\,\mathrm{mT}$, only one QD is on resonance while the other two are off-resonance. From the slope of the overlaid dashed lines we estimate a $g$-factor of $\approx 21$, 22 and 23 for the D1, D2, and D3, respectively ($E_{\mathrm{Z}} = g\mu_{\mathrm{B}}B$, where $\mu_{\mathrm{B}}$ is the Bohr magneton). We note that the $g$-factor can be affected by the coupling between the QDs and the neighbouring hybrids and that it might vary also as a function of the field. This can affect the estimation of the Zeeman energy. Therefore, it is important to verify that in the QD$_n$ spectra (panels **a-c**) there are no excited states that disperse with $\delta V_{\mathrm{D}n}$. This ensures that the Zeeman energy is larger than the scanned voltage bias range.

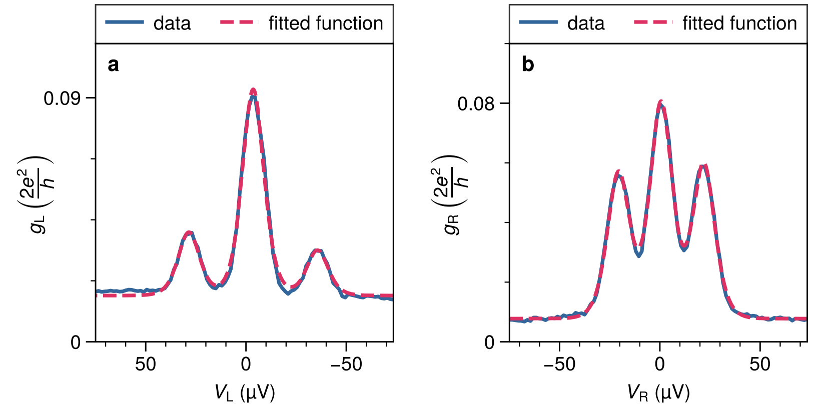


Fig. S8. **Fit of the two-site chain spectra of Fig. 2b,d.** Every peak is fitted with a cosh function as suggested in Refs. [5, 28], yielding $g_i(V_i) = A_1 \cosh\left(\frac{V_i - 2t_j}{\gamma}\right)^{-2} + A_2 \cosh\left(\frac{V_i}{\gamma}\right)^{-2} + A_3 \cosh\left(\frac{V_i + 2t_j}{\gamma}\right)^{-2} + B_i$ where $i \in \{\mathrm{L}, \mathrm{R}\}$ and $j \in \{1, 2\}$. We extract a $|2t_1| = |2\Delta_1| \approx 32\,\mathrm{\mu V}$ energy gap for the left two-site chain (panel **a**) and a $|2t_2| = |2\Delta_2| \approx 21\,\mathrm{\mu V}$ energy gap for the right two-site chain (panel **b**). Both panels yield a peak broadening $\gamma \approx 7\,\mathrm{\mu V}$. We note that there is a finite background conductance $B_{\mathrm{L}} \approx 0.017\frac{2e^2}{h}$ on the left and $B_{\mathrm{R}} \approx 0.008\frac{2e^2}{h}$ on the right, which we attribute to the capacitive response of the fridge lines to the lockin excitations [29].

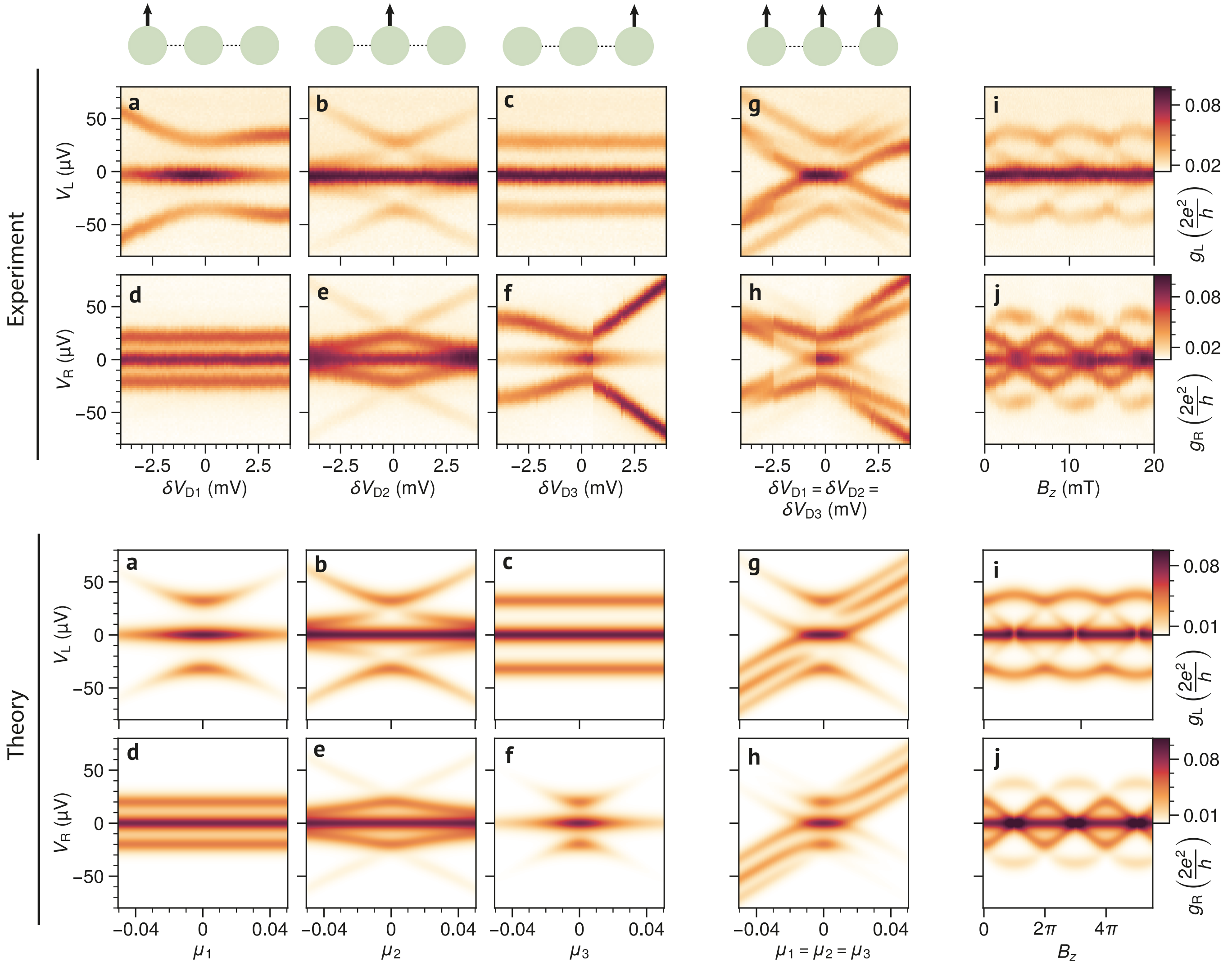


Fig. S9. **Experiment.** Conductance spectroscopy of the three-site chain presented in Figs. 2e and 3. **a-f.** The spectrum, measured from both the left and right lead, as a function of each QD making up the chain. In all panels, only a single excited state is observed unless either the D2 or the phase is detuned.**g, h.** The spectrum measured from the left and right lead, as a function of all QDs of the chain detuned simultaneously. Two excited states are visible when all QDs are detuned, yet only one excited state remains when all QDs are aligned at zero energy. **i, j.** The spectrum measured from the left and right lead, as a function of the out-of-plane field $B_z$. **Theory. a-j.** Corresponding theoretical simulations of the differential conductance of a three-site chain. Calculations are performed following the scattering matrix approach [18], assuming a finite temperature of $k_B T = 3\,\mu\text{eV}$ and a finite coupling to the normal leads: $\Gamma_L = \Gamma_R = 0.7\,\mu\text{eV}$, while using the extracted coupling amplitudes of Fig. S8; $|t_1| = |\Delta_1| = 16\,\mu\text{eV}$ and $|t_2| = |\Delta_2| = 10\,\mu\text{eV}$.

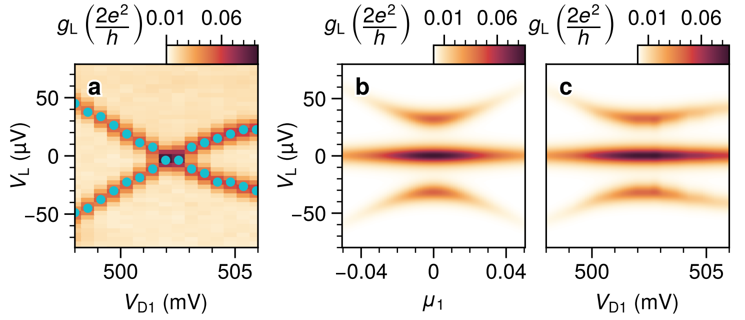


Fig. S10. **Example of a refined theoretical simulation. a.** D1 spectroscopy across one charge degeneracy point, measured with D2 and D3 about 5 mV off-resonance. The overlaid dotted lines extract the on-site energy $\mu_1(V_{D1})$. **b.** Theoretical simulation of the differential conductance spectrum of a three-site chain as a function of $\mu_1$, as in Fig. S9a. **c.** The same simulation of panel b, now displayed as a function of $V_{D1}$. If $\mu_1(V_{D1})$ were a linear function ($\mu_1 = \alpha_1 e \delta V_{D1}$, where $\alpha_1$ is the lever-arm and $e$ the electron charge), then panels b and c would look identical.

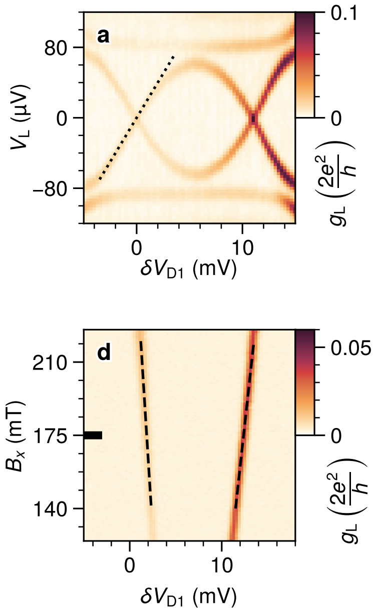


Fig. S11. **QD characterization of sweet spot B. a-c.** Conductance spectroscopy as a function of $V_{D1}, V_{D2}$, and $V_{D3}$. Each panel shows a single QD orbital, measured at $B_x = 175\,\mathrm{mT}$. $\delta V_{Dn} = 0$ denotes the QD resonance making up the Kitaev chain for sweet spot B, corresponding to a spin configuration of; $\downarrow, \uparrow, \downarrow$. The lever arm of each QD plunger gate is estimated from the slope of the overlaid dotted lines, corresponding to $\alpha_1, \alpha_{2\downarrow}, \alpha_{2\uparrow}, \alpha_3 = 0.020, 0.08, 0.012, 0.015$ for $D1$, the left resonance of $D2$, the right resonance of $D2$, and $D3$, respectively. **d-f.** Zero-bias conductance as a function of $B_x$, the field along the nanowire, and $V_{D1}, V_{D2}, V_{D3}$, respectively. From the slope of the overlaid dashed lines we estimate a $g$-factor of $\approx$ 15, 25 and 20 for D1, D2, and D3, respectively.

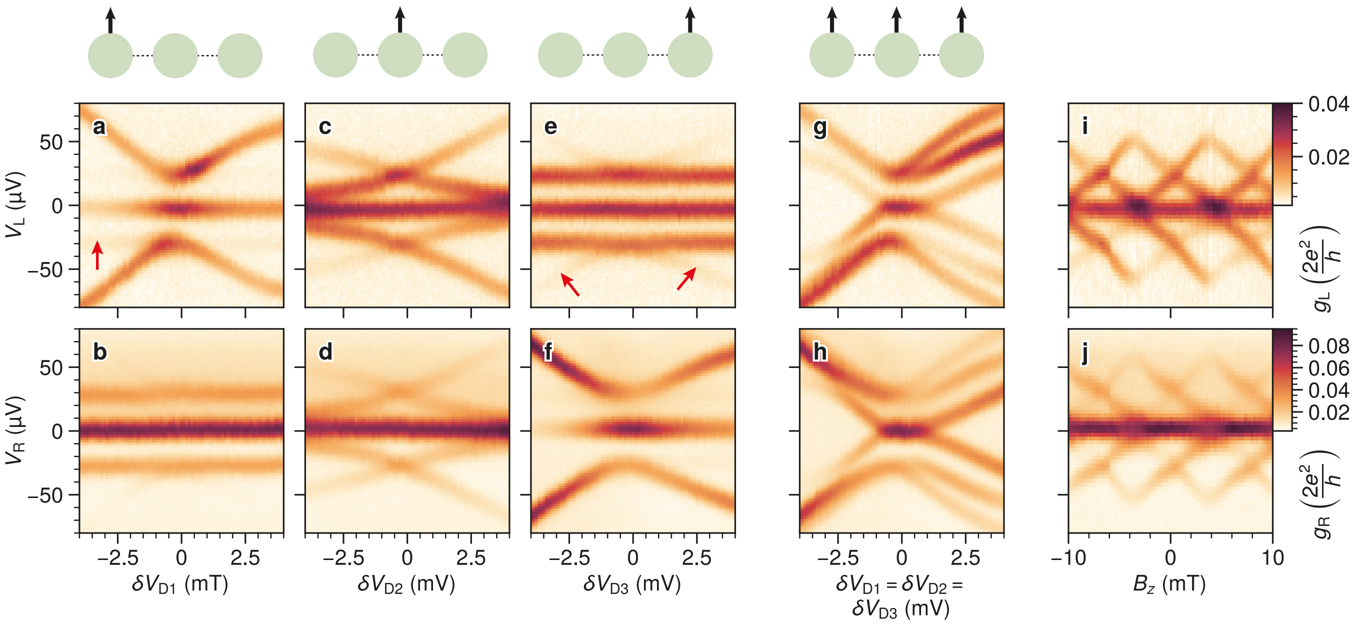


Fig. S12. **Characterization of the three-site chain spectra in sweet spot B, reproduction of Fig. 3. a-f.** The spectrum, measured from the left and right lead, as a function of each QD making up the chain. We note that in some panels a second excited state is faintly visible (see red arrows in panels **a** and **e**). This could be due to the lower Zeeman energy (compare Fig. S11 and S7) possibly enabling next-nearest-neighbour coupling or to a less precise identification of the field corresponding to $\varphi = 0$, here set at $B_z = 0.8\,\mathrm{mT}$. **g, h.** The spectrum measured from the left and right lead, as a function of all QDs of the chain detuned simultaneously. **i, j.** The spectrum measured from the left and right lead, as a function of the out-of-plane field $B_z$.

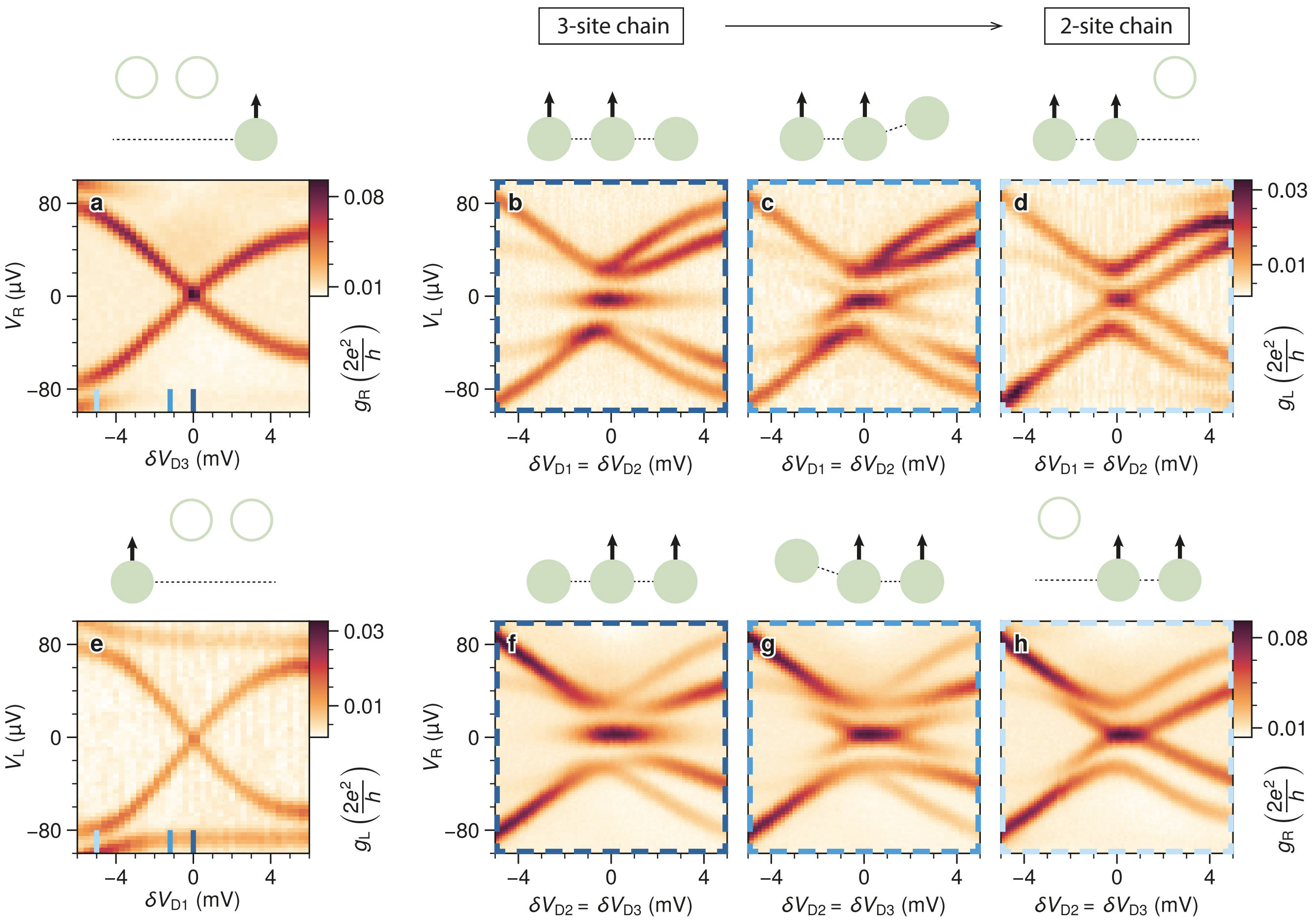


Fig. S13. **Transition from a three-site chain to a two-site chain as one quantum dot is detuned.** Here we show the measured conductance spectra for sweet spot B for different detunings of the outer QD, demonstrating the ability to tune from the three-site to the two-site regime by moving one of the outer QDs off-resonance. **a.** Conductance spectroscopy $g_{\mathrm{R}}$, measured from the right lead as a function of $V_{\mathrm{D3}}$, revealing the sub-gap density of states of D3. The panel is centered around a single charge degeneracy point. D1 and D2 were placed off-resonance during this measurement. Blue insets denote the position of $V_{\mathrm{D3}}$ in subsequent panels **b**, **c** and **d**. **b-d.** The spectrum of the chain measured from the left lead as a function of $V_{\mathrm{D1}} = V_{\mathrm{D2}}$ at three different positions of $V_{\mathrm{D3}}$. In panel **b**, D3 is positioned on resonance, restoring the three-site chain when $\delta V_{\mathrm{D1}} = \delta V_{\mathrm{D2}} = 0$. This is evident from the persistent zero-bias peak upon detuning $\delta V_{\mathrm{D1}}$ and $\delta V_{\mathrm{D2}}$. In panel **c**, $V_{\mathrm{D3}}$ is slightly detuned. The zero-bias peak is now split when $\delta V_{\mathrm{D1}}$ and $\delta V_{\mathrm{D2}}$ are detuned. Finally, in panel **d**, $V_{\mathrm{D3}}$ is placed off-resonance. Judging from panel **a**, its chemical potential is now $\approx 80\,\mu\mathrm{V}$ ($> |t_2| = |\Delta_2|$) such that it no longer plays a significant role in the low-energy spectrum of the chain. Therefore, we argue that the chain at that point can be approximated as a two-site chain. **e-h.** The same experiment performed from the opposite side of the chain. When placing $V_{\mathrm{D1}}$ far off-resonance (panel **h**), the right side of the device converts to a two-site chain.

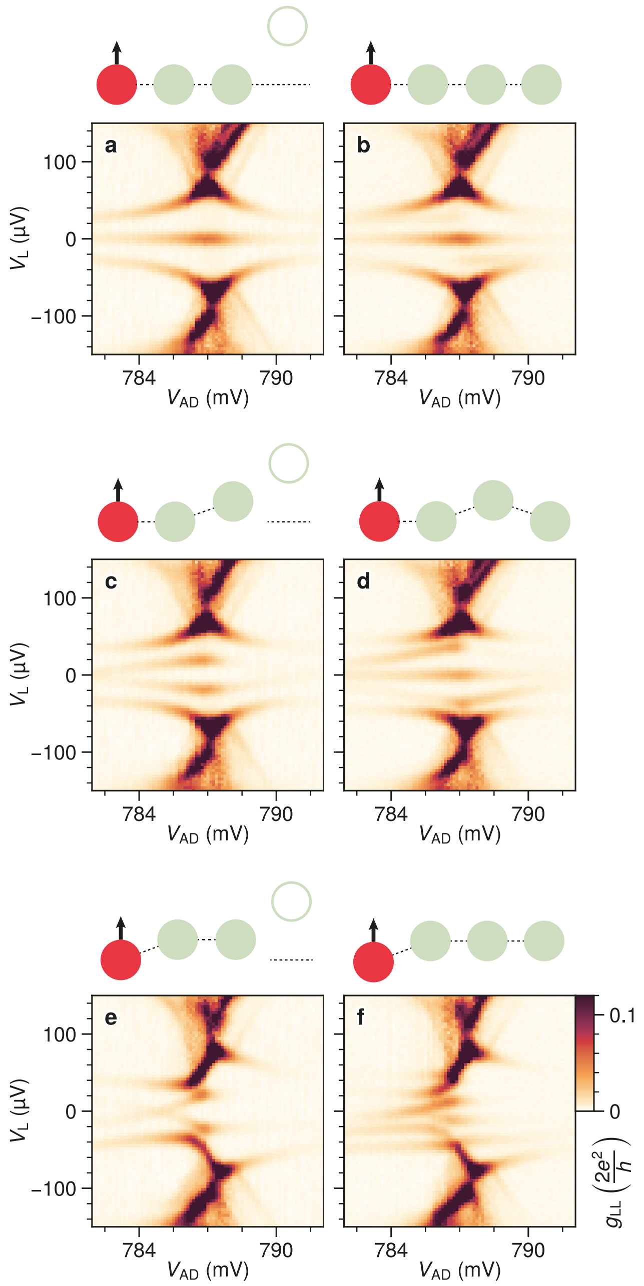


Fig. S14. **QD-test for sweet spot B, reproduction of Fig. 4. a-b.** QD-test at the sweet spot for the two-site and three-site chain, respectively, similar to Fig. 4c,g. **c-f.** QD-test for the detuned two-site and three-site chain, resembling Fig. 4b,f,a,e, respectively.

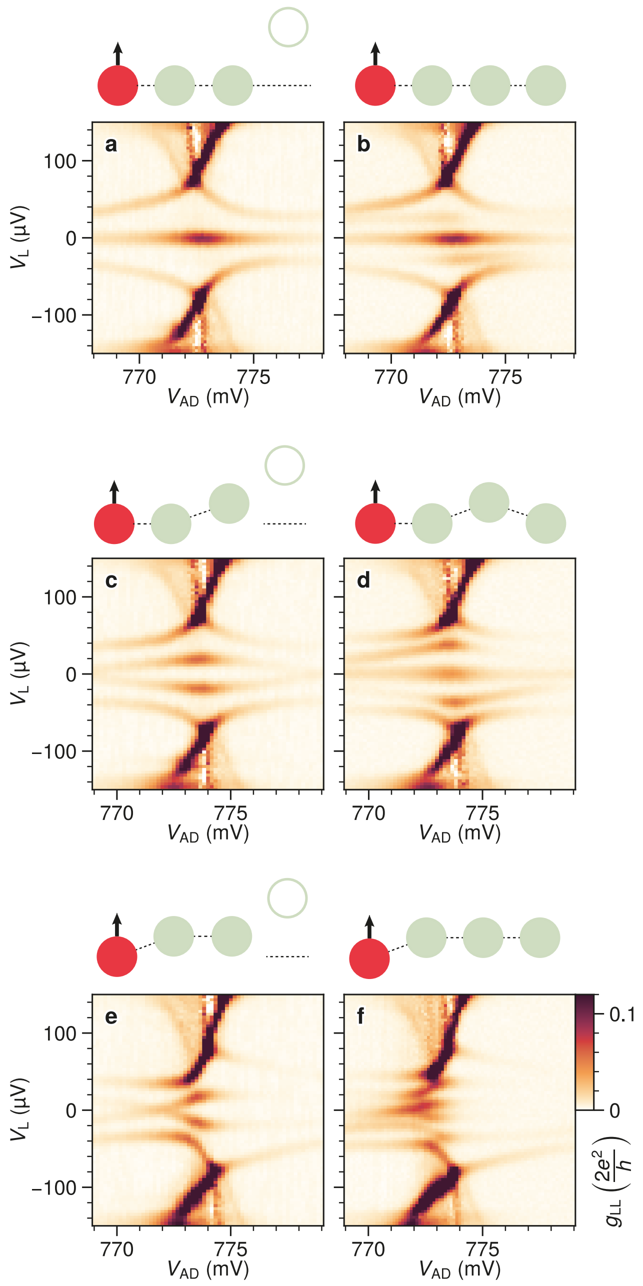


Fig. S15. **QD-test for sweet spot B with altered spin configuration.** Same as Fig. S14 but using a different spin in the additional quantum dot.

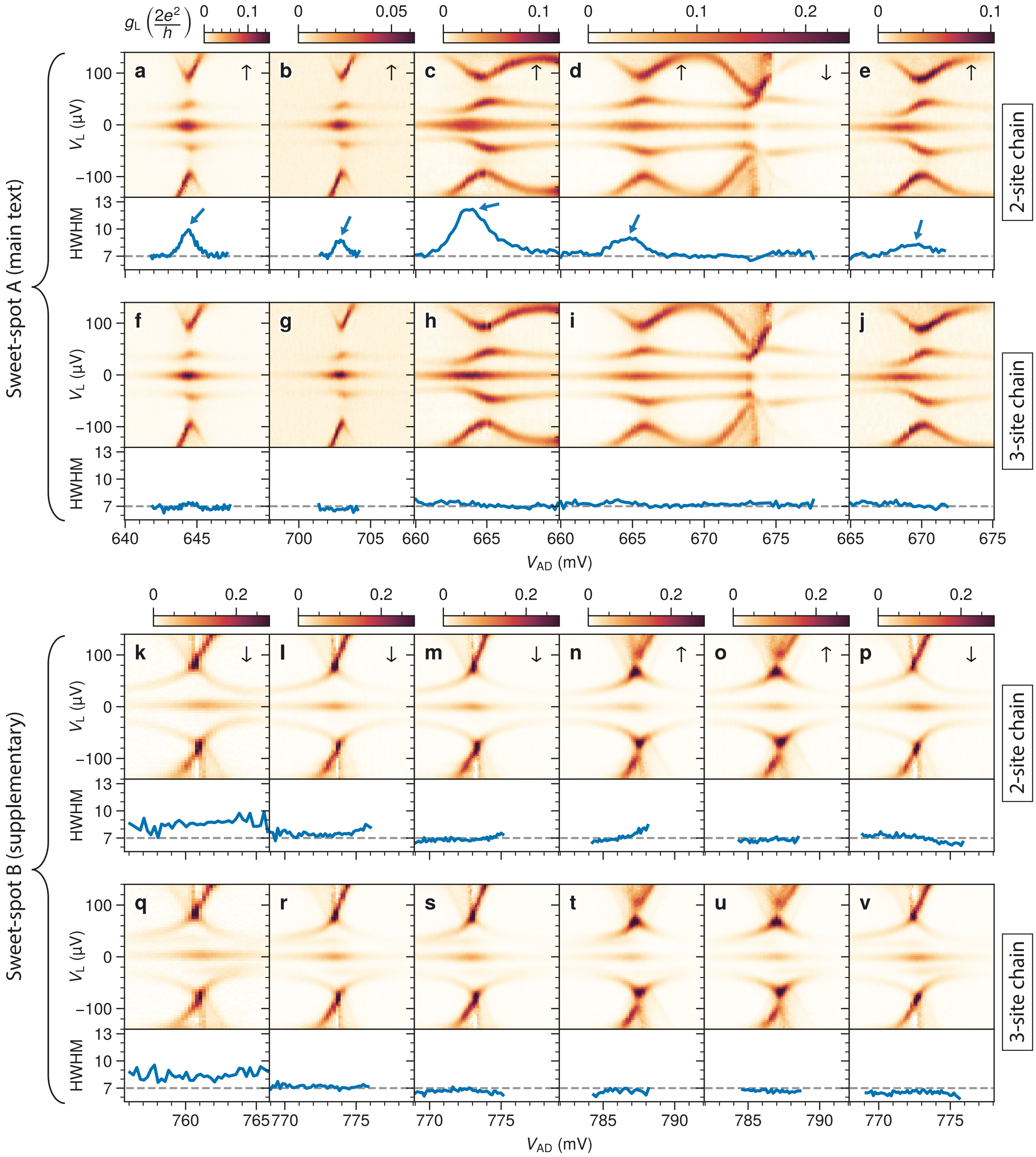


Fig. S16. **Inventory of all the QD-tests comparing two- and three-site chains.** Here we report all measured QD-tests comparing two- and three-site chains at the sweet-spot. The first two rows are measured on sweet spot A (studied in Figs. 1 to 4 and S7 to S9) whilst the last two rows are measured on the sweet spot B (Figs. S11 to S15). Even rows study three-site chains while odd rows study the corresponding two-site chains after setting D3 off-resonance. For each sweet spot, multiple iterations of the QD-test were performed. Each iteration corresponds to a different tune-up of the additional quantum dot or to a fine adjustment in the sweet-spot centering. Panels **e** and **j** report the same data of Fig. 4c,g; panels **o** and **u** report the same data of Fig. S14a,b; panels **p** and **v** report the same data of Fig. S15a,b. For every QD-test, the black arrow in the top-right corner indicates the electron spin in the additional QD, it is inferred from the comparison with the theory model.
Below every conductance spectrum, we plot the half-width at half-maximum (HWHM) of the zero-bias peak (ZBP), wherever the ZBP height is at least $0.01\frac{2e^2}{h}$ (otherwise the signal is too low to extract the HWHM reliably). Whenever the additional quantum dot is off-resonance, we measure HWHM $= 7 \pm 1\,\mu$V, apart from panels **k** and **q** where it is slightly larger due to lower $V_L$ resolution (see code in the linked repository [3]). Conversely, when AD is brought into resonance, we sometimes resolve a different behaviour for two- and three-site chains: the latter always show HWHM $= 7 \pm 1\,\mu$V, whereas for two-site chains there is sometimes an excess in the measured HWHM, highlighted by the blue arrows. We attribute this excess in the measured HWHM to an imperfect centering at the sweet spot.

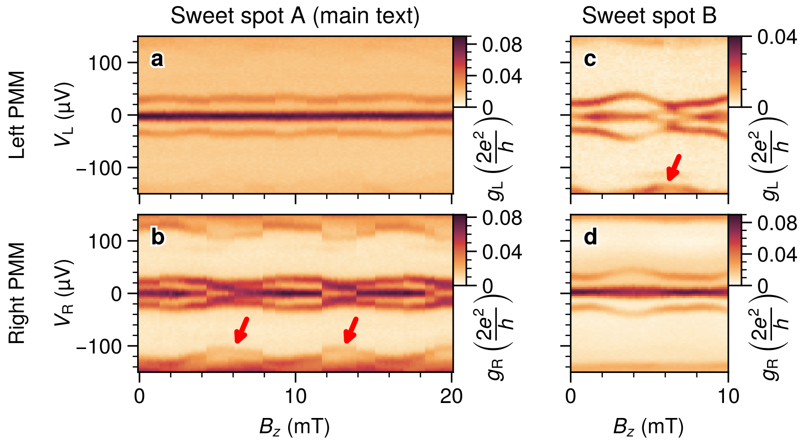


Fig. S17. **Phase dependence of two-site Kitaev chains.** The left column (panels **a** and **b**) concerns sweet spot A (discussed in Figs. 1 to 4 and S7 to S9); the second column (panels **c** and **d**) concerns sweet spot B (characterized in Figs. S11 to S15). In the first row, D3 is set off-resonance to define a two-site chain on the left side of the device. In the second row, D1 is set off-resonance (with D3 back on resonance), to define a two-site chain on the right side. We note that the measured two-site conductance spectra show some phase dependence. This could be attributed to the corresponding modulation of the energy of the Andreev bound states (ABSs) populating the hybrid regions [30]. They are marked with red arrows in panel **b** and **c**. Since the ABSs influence the $t_n$ and $\Delta_n$ couplings [31–34], their modulation can affect the $|t_n| = |\Delta_n|$ sweet-spot condition (see for instance panel **c**, where the ZBP is split at $\approx 6\,\mathrm{mT}$). Hence, the sweet-spot condition of three-site chains might be slightly imprecise if $B_z$ is varied (Figs. 2e,f,g and S18). When $B_z$ is fixed (all other figures), we optimise for an accurate $|t_n| = |\Delta_n|\ \forall n$.

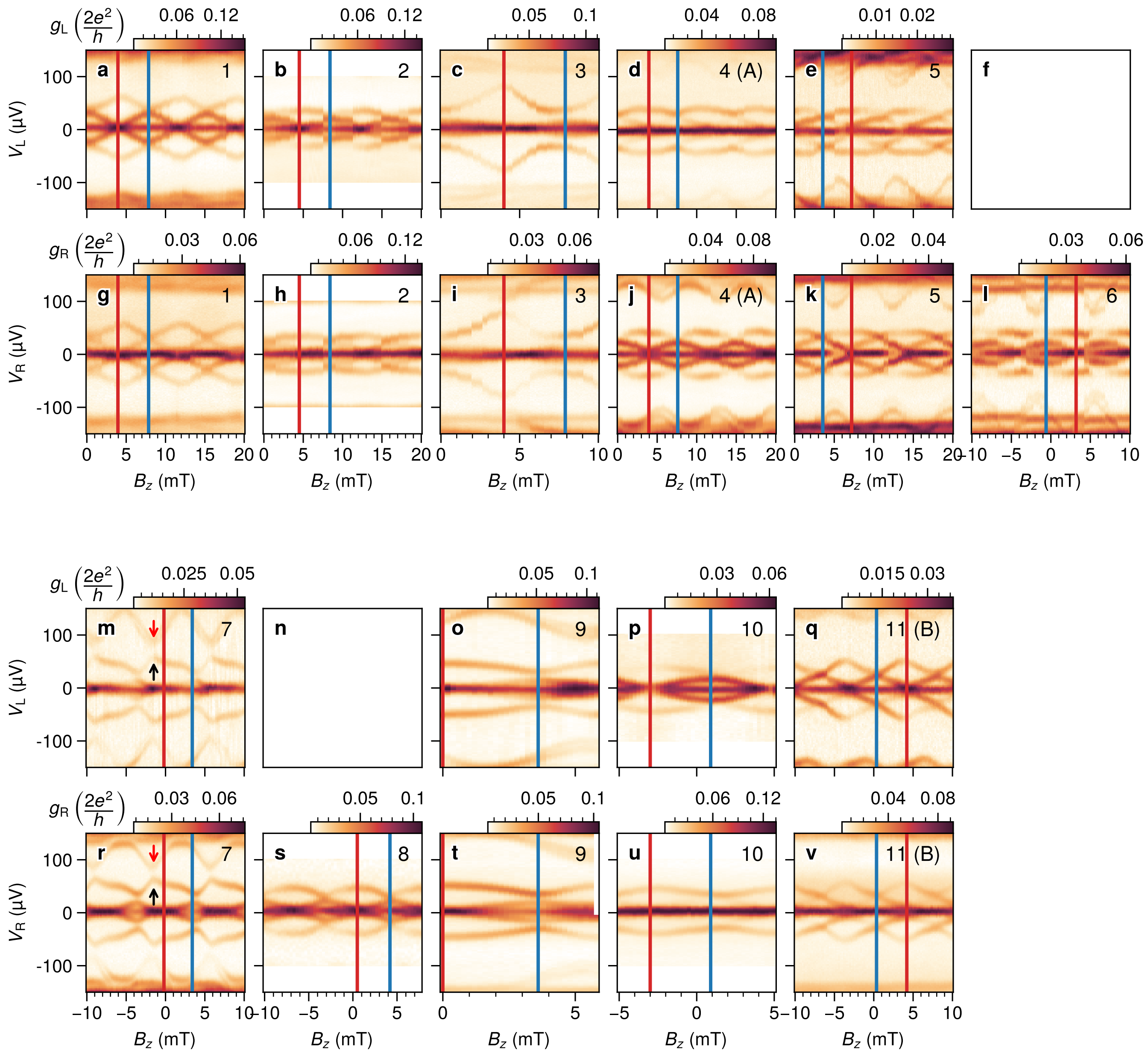


Fig. S18. **Full catalog of three-site Kitaev chain spectra as a function of $B_z$.** Overall, we measured the $B_z$ dependence of 11 different three-site sweet spots; they are labelled with the numbers in the top left corner of each panel. Different sweet spots correspond to different tune-ups of the device, hence, they can differ in both the QD orbitals and the hybrid settings. Different sweet spots were characterized to different extents, for instance, we performed QD-tests only on sweet spots 4 (i.e. "A") and 11 (i.e. "B"). Panels **f** and **n** are white because we did not measure the left conductance in those cases. In all other panels, blue lines identify the field where $\varphi = 0$, red lines identify the field where $\varphi = \pi$. Here, in contrast to Fig. 2, the 0 and $\pi$ points are identified manually by comparing the measured spectra with simulations (see Fig. S9i,j). The identification was repeated, independently, by two operators, yielding an average difference of 0.2 mT (see code in the linked repository [3]). We note that some of the spectra appear distorted. For instance, in panels **m** and **r** the second excited state appears anomalously high in energy (black arrow) near the minimum ABS energy (red arrows). This is consistent with what is discussed in Fig. S17: a lower ABS energy can increase the $t_n$ and $\Delta_n$ amplitudes [32]. Finally, we note that the period is $\approx$ 7.5 mT for all 11 cases, corresponding to a loop area of $\approx 0.28\,\mu\text{m}^2$. However, the internal area of our superconducting loop is $\approx 0.13\,\mu\text{m}^2$. The discrepancy suggests that the field penetrates the Al film.

[1] A. Tsintzis, R. S. Souto, and M. Leijnse, Creating and detecting poor man's Majorana bound states in interacting quantum dots, Physical Review B **106**, 10.1103/physrevb.106.l201404 (2022).

[2] R. A. Dourado, M. Leijnse, and R. S. Souto, Majorana sweet spots in three-site kitaev chains, Physical Review B **111**, 10.1103/physrevb.111.235409 (2025).

[3] F. J. Bennebroek Evertsz', A. Bordin, and J. D. Torres Luna, Probing Majorana localization of a phase-controlled three-site Kitaev chain with an additional quantum dot (2025).

[4] P. Virtanen, R. Gommers, T. E. Oliphant, M. Haberland, T. Reddy, D. Cournapeau, E. Burovski, P. Peterson, W. Weckesser, J. Bright, S. J. van der Walt, M. Brett, J. Wilson, K. J. Millman, N. Mayorov, A. R. J. Nelson, E. Jones, R. Kern, E. Larson, C. J. Carey, İ. Polat, Y. Feng, E. W. Moore, J. VanderPlas, D. Laxalde, J. Perktold, R. Cimrman, I. Henriksen, E. A. Quintero, C. R. Harris, A. M. Archibald, A. H. Ribeiro, F. Pedregosa, P. van Mulbregt, and SciPy 1.0 Contributors, SciPy 1.0: Fundamental Algorithms for Scientific Computing in Python, Nature Methods **17**, 261 (2020).

[5] S. L. D. ten Haaf, Y. Zhang, Q. Wang, A. Bordin, C.-X. Liu, I. Kulesh, V. P. M. Sietses, C. G. Prosko, D. Xiao, C. Thomas, M. J. Manfra, M. Wimmer, and S. Goswami, Observation of edge and bulk states in a three-site kitaev chain, Nature **641**, 890–895 (2025).

[6] V. Mourik, K. Zuo, S. M. Frolov, S. R. Plissard, E. P. A. M. Bakkers, and L. P. Kouwenhoven, Signatures of Majorana Fermions in Hybrid Superconductor-Semiconductor Nanowire Devices, Science **336**, 1003–1007 (2012).

[7] S. Vaitiekėnas, G. W. Winkler, B. van Heck, T. Karzig, M.-T. Deng, K. Flensberg, L. I. Glazman, C. Nayak, P. Krogstrup, R. M. Lutchyn, and C. M. Marcus, Flux-induced topological superconductivity in full-shell nanowires, Science **367**, 10.1126/science.aav3392 (2020).

[8] M. Aghaee, A. Akkala, Z. Alam, R. Ali, A. Alcaraz Ramirez, M. Andrzejczuk, A. E. Antipov, P. Aseev, M. Astafev, B. Bauer, J. Becker, S. Boddapati, F. Boekhout, J. Bommer, T. Bosma, L. Bourdet, S. Boutin, P. Caroff, L. Casparis, M. Cassidy, S. Chatoor, A. W. Christensen, N. Clay, W. S. Cole, F. Corsetti, A. Cui, P. Dalampiras, A. Dokania, G. de Lange, M. de Moor, J. C. Estrada Saldaña, S. Fallahi, Z. H. Fathabad, J. Gamble, G. Gardner, D. Govender, F. Griggio, R. Grigoryan, S. Gronin, J. Gukelberger, E. B. Hansen, S. Heedt, J. Herranz Zamorano, S. Ho, U. L. Holgaard, H. Ingerslev, L. Johansson, J. Jones, R. Kallaher, F. Karimi, T. Karzig, E. King, M. E. Kloster, C. Knapp, D. Kocon, J. Koski, P. Kostamo, P. Krogstrup, M. Kumar, T. Laeven, T. Larsen, K. Li, T. Lindemann, J. Love, R. Lutchyn, M. H. Madsen, M. Manfra, S. Markussen, E. Martinez, R. McNeil, E. Memisevic, T. Morgan, A. Mullally, C. Nayak, J. Nielsen, W. H. P. Nielsen, B. Nijholt, A. Nurmohamed, E. O'Farrell, K. Otani, S. Pauka, K. Petersson, L. Petit, D. I. Pikulin, F. Preiss, M. Quintero-Perez, M. Rajpalke, K. Rasmussen, D. Razmadze, O. Reentila, D. Reilly, R. Rouse, I. Sadovskyy, L. Sainiemi, S. Schreppler, V. Sidorkin, A. Singh, S. Singh, S. Sinha, P. Sohr, T. Stankevič, L. Stek, H. Suominen, J. Suter, V. Svidenko, S. Teicher, M. Temuerhan, N. Thiyagarajah, R. Tholapi, M. Thomas, E. Toomey, S. Upadhyay, I. Urban, S. Vaitiekėnas, K. Van Hoogdalem, D. Van Woerkom, D. V. Viazmitinov, D. Vogel, S. Waddy, J. Watson, J. Weston, G. W. Winkler, C. K. Yang, S. Yau, D. Yi, E. Yucelen, A. Webster, R. Zeisel, and R. Zhao, InAs-Al hybrid devices passing the topological gap protocol, Physical Review B **107**, 10.1103/physrevb.107.245423 (2023).

[9] R. Hess, H. F. Legg, D. Loss, and J. Klinovaja, Trivial Andreev Band Mimicking Topological Bulk Gap Reopening in the Nonlocal Conductance of Long Rashba Nanowires, Physical Review Letters **130**, 10.1103/physrevlett.130.207001 (2023).

[10] E. J. H. Lee, X. Jiang, R. Aguado, G. Katsaros, C. M. Lieber, and S. De Franceschi, Zero-Bias Anomaly in a Nanowire Quantum Dot Coupled to Superconductors, Physical Review Letters **109**, 10.1103/physrevlett.109.186802 (2012).

[11] A. Vuik, B. Nijholt, A. Akhmerov, and M. Wimmer, Reproducing topological properties with quasi-Majorana states, SciPost Physics **7**, 10.21468/scipostphys.7.5.061 (2019).

[12] R. M. Lutchyn, J. D. Sau, and S. Das Sarma, Majorana Fermions and a Topological Phase Transition in Semiconductor-Superconductor Heterostructures, Physical Review Letters **105**, 10.1103/physrevlett.105.077001 (2010).

[13] Y. Oreg, G. Refael, and F. von Oppen, Helical Liquids and Majorana Bound States in Quantum Wires, Physical Review Letters **105**, 10.1103/physrevlett.105.177002 (2010).

[14] E. Prada, P. San-Jose, M. W. A. de Moor, A. Geresdi, E. J. H. Lee, J. Klinovaja, D. Loss, J. Nygård, R. Aguado, and L. P. Kouwenhoven, From Andreev to Majorana bound states in hybrid superconductor–semiconductor nanowires, Nature Reviews Physics **2**, 575–594 (2020).

[15] S. L. D. ten Haaf, Q. Wang, A. M. Bozkurt, C.-X. Liu, I. Kulesh, P. Kim, D. Xiao, C. Thomas, M. J. Manfra, T. Dvir, M. Wimmer, and S. Goswami, A two-site Kitaev chain in a two-dimensional electron gas, Nature **630**, 329–334 (2024).

[16] A. M. Bozkurt, S. Miles, S. L. D. ten Haaf, C.-X. Liu, F. Hassler, and M. Wimmer, Interaction-induced strong zero modes in short quantum dot chains with time-reversal symmetry, SciPost Physics **18**, 10.21468/scipostphys.18.6.206 (2025).

[17] M. Luethi, H. F. Legg, D. Loss, and J. Klinovaja, From perfect to imperfect poor man's Majoranas in minimal Kitaev chains, Physical Review B **110**, 10.1103/physrevb.110.245412 (2024).

[18] T. Dvir, G. Wang, N. van Loo, C.-X. Liu, G. P. Mazur, A. Bordin, S. L. D. ten Haaf, J.-Y. Wang, D. van Driel, F. Zatelli, X. Li, F. K. Malinowski, S. Gazibegovic, G. Badawy, E. P. A. M. Bakkers, M. Wimmer, and L. P. Kouwenhoven, Realization of a minimal Kitaev chain in coupled quantum dots, Nature **614**, 445–450 (2023).

[19] R. Koch, D. van Driel, A. Bordin, J. L. Lado, and E. Greplova, Adversarial Hamiltonian learning of quantum dots in a minimal Kitaev chain, Physical Review Applied **20**, 10.1103/physrevapplied.20.044081 (2023).

[20] F. Zatelli, D. van Driel, D. Xu, G. Wang, C.-X. Liu, A. Bordin, B. Roovers, G. P. Mazur, N. van Loo, J. C. Wolff, A. M. Bozkurt, G. Badawy, S. Gazibegovic, E. P. A. M. Bakkers, M. Wimmer, L. P. Kouwenhoven, and T. Dvir, Robust poor man's Majorana zero modes using Yu-Shiba-Rusinov states, Nature Communications **15**, 10.1038/s41467-024-52066-2

(2024).

[21] D. van Driel, R. Koch, V. P. M. Sietses, S. L. D. ten Haaf, C.-X. Liu, F. Zatelli, B. Roovers, A. Bordin, N. van Loo, G. Wang, J. C. Wolff, G. P. Mazur, T. Dvir, I. Kulesh, Q. Wang, A. M. Bozkurt, S. Gazibegovic, G. Badawy, E. P. A. M. Bakkers, M. Wimmer, S. Goswami, J. L. Lado, L. P. Kouwenhoven, and E. Greplova, Cross-Platform Autonomous Control of Minimal Kitaev Chains (2024).

[22] A. Bordin, C.-X. Liu, T. Dvir, F. Zatelli, S. L. D. ten Haaf, D. van Driel, G. Wang, N. van Loo, Y. Zhang, J. C. Wolff, T. Van Caekenberghe, G. Badawy, S. Gazibegovic, E. P. A. M. Bakkers, M. Wimmer, L. P. Kouwenhoven, and G. P. Mazur, Enhanced majorana stability in a three-site kitaev chain, Nature Nanotechnology 10.1038/s41565-025-01894-4 (2025).

[23] N. van Loo, F. Zatelli, G. O. Steffensen, B. Roovers, G. Wang, T. Van Caekenberghe, A. Bordin, D. van Driel, Y. Zhang, W. D. Huisman, G. Badawy, E. P. A. M. Bakkers, G. P. Mazur, R. Aguado, and L. P. Kouwenhoven, Single-shot parity readout of a minimal kitaev chain (2025).

[24] L. Kouwenhoven, Perspective on Majorana bound-states in hybrid superconductor-semiconductor nanowires, Modern Physics Letters B 10.1142/s0217984925400020 (2024).

[25] J. D. Sau and S. Das Sarma, Realizing a robust practical Majorana chain in a quantum-dot-superconductor linear array, Nature Communications **3**, 10.1038/ncomms1966 (2012).

[26] P. Stano and D. Loss, Review of performance metrics of spin qubits in gated semiconducting nanostructures, Nature Reviews Physics **4**, 672–688 (2022).

[27] M. Kjaergaard, M. E. Schwartz, J. Braumüller, P. Krantz, J. I.-J. Wang, S. Gustavsson, and W. D. Oliver, Superconducting Qubits: Current State of Play, Annual Review of Condensed Matter Physics **11**, 369–395 (2020).

[28] C. W. J. Beenakker, Theory of coulomb-blockade oscillations in the conductance of a quantum dot, Physical Review B **44**, 1646–1656 (1991).

[29] Q. Wang, Y. Zhang, S. Karwal, and S. Goswami, Spatial Dependence of Local Density of States in Semiconductor-Superconductor Hybrids, Nano Letters **24**, 13558–13563 (2024).

[30] A. Bordin, F. J. Bennebroek Evertsz', G. O. Steffensen, T. Dvir, G. P. Mazur, D. van Driel, N. van Loo, J. C. Wolff, E. P. Bakkers, A. L. Yeyati, and L. P. Kouwenhoven, Impact of Andreev Bound States within the Leads of a Quantum Dot Josephson Junction, Physical Review X **15**, 10.1103/physrevx.15.011046 (2025).

[31] C.-X. Liu, G. Wang, T. Dvir, and M. Wimmer, Tunable Superconducting Coupling of Quantum Dots via Andreev Bound States in Semiconductor-Superconductor Nanowires, Physical Review Letters **129**, 10.1103/physrevlett.129.267701 (2022).

[32] A. Bordin, G. Wang, C.-X. Liu, S. L. D. ten Haaf, N. van Loo, G. P. Mazur, D. Xu, D. van Driel, F. Zatelli, S. Gazibegovic, G. Badawy, E. P. A. M. Bakkers, M. Wimmer, L. P. Kouwenhoven, and T. Dvir, Tunable Crossed Andreev Reflection and Elastic Cotunneling in Hybrid Nanowires, Physical Review X **13**, 10.1103/physrevx.13.031031 (2023).

[33] J. D. Torres Luna, A. M. Bozkurt, M. Wimmer, and C.-X. Liu, Flux-tunable Kitaev chain in a quantum dot array, SciPost Physics Core **7**, 10.21468/scipostphyscore.7.3.065 (2024).

[34] I. Kulesh, S. L. D. ten Haaf, Q. Wang, V. P. M. Sietses, Y. Zhang, S. R. Roelofs, C. G. Prosko, D. Xiao, C. Thomas, M. J. Manfra, and S. Goswami, Flux-controlled two-site kitaev chain, Physical Review Letters **135**, 10.1103/r9pv-2prs (2025).
